# Single Nucleotide Variants as Proxies for HLA-A*31:01 in Native American Populations

**DOI:** 10.3389/fphar.2022.849136

**Published:** 2022-04-14

**Authors:** Vanessa Câmara Fernandes, Marco Antônio M. Pretti, Luiza Tamie Tsuneto, Maria Luiza Petzl-Erler, Guilherme Suarez-Kurtz

**Affiliations:** ^1^ Coordenação de Pesquisa, Instituto Nacional de Câncer, Rio de Janeiro, Brazil; ^2^ Laboratório de Bioinformática e Biologia Computacional, Instituto Nacional de Câncer, Rio de Janeiro, Brazil; ^3^ Departamento de Análises Clínicas, Universidade Estadual de Maringá, Maringá, Brazil; ^4^ Programa de Pós-Graduação em Genética, Laboratório de Genética Molecular Humana, Departamento de Genética, Universidade Federal do Paraná, Curitiba, Brazil

**Keywords:** carbamazepine, HLA-A*31:01, idiosyncratic reactions, rs1061235, rs17179220

## Abstract

Carbamazepine triggers dermatologic hypersensitivity reactions, associated with specific human leukocyte antigens (HLAs), especially HLA-B*15:02 and HLA-A*31:01. Previous efforts to identify single nucleotide variants (SNVs) with high predictive value as HLA proxies, revealed that rs1061235 and rs17179220 fulfill these requirements for HLA-A*31:01 in some but not all populations. Herein we explored the predictive performance of rs1061235 and rs17179220 as HLA-A*31:01 tags in populations of Native American ancestry, which are largely underrepresented in pharmacogenomic studies. The study cohorts comprised the overall Admixed American superpopulation of the 1000 Genomes Project (1 KG_AMR), a subcohort of individuals with predominant Native American ancestry (1 KG_NAT), the Native American population of the Human Genome Diversity Project (HGDP), plus Kaingang (KRC) and Guarani (GRC and GKW) adults from indigenous reservation areas in Brazil. The diversity of cohorts is reflected in the range of frequencies of HLA-A*31:01 (0.02–0.65), rs1061235 (0.03–0.13) and rs17179220 (0.12–0.66), as well as in the predictive performance of these SNVs as HLA-A*31:01 proxies. NPV (negative predictive value), the metric of primary interest for pharmacogenetic-informed carbamazepine prescription was maximal (NPV = 1.0) for both SNVs in 1 KG_AMR and 1 KG_NAT, for rs17179220, but not rs1061235 (NPV = 0.91) in HGDP, and for rs17179220 in GWK, but not GRC (NPV = 0.88) or KRC (NPV = 0.80). Collectively, the data support the notion that rs1061235 and rs17179220 are not optimal proxies for HLA-A*31:01 across populations of Native American ancestry.

## Introduction

Carbamazepine, an effective anticonvulsant drug, also used to treat bipolar disorder and to relieve pain in trigeminal neuralgia, is known to trigger hypersensitivity reactions that typically affect the skin. While some of these reactions are mild, other conditions such as Stevens-Johnson syndrome (SJS), toxic epidermal necrolysis (TEN), and drug reactions with eosinophilia and systemic symptoms (DRESS) are potentially life-threatening. There is strong evidence of genetic predisposition for these idiosyncratic reactions, linked to specific human leukocyte antigen (HLA) types, especially HLA-B*15:02 in Southeast Asian populations and HLA-A*31:01 in more widespread regions (Dean et al., 2015; Yip and Pirmohamed, 2017). Accordingly, the Clinical Pharmacogenetics Implementation Consortium (CPIC) and the Dutch Pharmacogenetics Working Group (DWPG) dosing guidelines for carbamazepine recommend the choice of an alternative drug for carbamazepine-naive patients carrying at least one copy of either HLA-B*15:02 or HLA-A*31:01 (Phillips et al., 2018; Dutch Pharmacogenetic Working Group, 2021). Recommendations for genetic testing for HLA-B*15:02 and/or HLA-A*31:01 for carbamazepine-naive patients before they start treatment have also been issued by the Canadian Pharmacogenomics Network for Drug Safety (Amstutz et al., 2014) and the United States Food and Drug Administration (Food and Drug Administration, 2021).

The fact that “gold-standard” methods for HLA haplotyping are time-consuming and relatively expensive, prompted efforts to validate single nucleotide variants (SNVs), as reliable tags for HLAs linked to idiosyncratic carbamazepine reactions. Two SNVs, namely rs1061235 (GRCh38.p13 chr 6, NC_000006.12:g.29945521A>T) and rs17179220 (GRCh38.p13 chr 6, NC_000006.12:g.29853458G>A) have been proposed for tagging the HLA-A*31:01 haplotype: de Bakker et al. (2006) first reported complete linkage disequilibrium (LD) between rs1061235 and HLA-A*31:01 in the HapMap European (CEU) sample. Subsequent work revealed that rs1061235 is also linked to HLA-A*33 haplotypes, which impacts the predictive performance of rs1061235 to identify carriers of HLA-A*31:01 (Amstutz et al., 2013; Thorstensen et al., 2014; Buchner et al., 2021). High LD of rs17179220 with HLA-A*31:01 was initially detected in Han Chinese (Zhou et al., 2016), and subsequently verified in a large set of ancestrally diverse populations (Erlichster et al., 2019).

The present study explores the reliability of rs1061235 and rs17179220 for tagging HLA-A*31:01 in populations with Native American ancestry. Two previous studies included individuals with Native American ancestry: Amstutz et al. (2013) enrolled five Canadian Aboriginal children whereas Erlichster et al. (2019) examined a subset of samples from the Admixed American superpopulation of the 1000 Genomes Project [1 KG_AMR; (1000 Genomes Project Consortium et al., 2015)]. Our study covers the overall 1 KG_AMR cohort, as well as Native American individuals from the Human Genome Diversity Project (HGDP) (Cavalli-Sforza, 2005) and from three groups living in indigenous reservation areas of Brazil, that differ largely in frequency of HLA haplotypes (Belich et al., 1992; Petzl-Erler et al., 1993; Parham et al., 1997). We did not extend our study to tags for HLA-B*15:02, the other major HLA haplotype linked to carbamazepine-induced idiosyncratic reactions, because this haplotype is rare or absent in our study cohorts.

## Methods

### Study Populations

The 1 KG_AMR (*n* = 347) superpopulation includes individuals from the South American countries Colombia (CLM, *n* = 94) and Peru (PEL, 85), from Puerto Rico (PUR, 104) and individuals with Mexican ancestry living in Los Angeles, USA (MXL, 64). Based on individual estimates of Native, European and African ancestry a subcohort designated 1 KG_NAT was devised, comprising the 80 individuals with the highest Native ancestry (Suarez-Kurtz et al., 2020). This subcohort includes 67 PEL, 12 MXL and one CLM, all with >70% Native ancestry (average 85.9%, 95% CI 85.9–88.2). The HGDP cohort (*n* = 61) is formed by samples of Native American groups, from Brazil (Surui and Karitiana), Mexico (Maya and Pima) and Colombia. Samples from Native populations living in indigenous reservation areas in Brazil were obtained from Kaingang (KRC) and Guarani (GRC and GKW) adults, previously enrolled in a study of population genetics of Brazilian Amerindians, approved by the Brazilian National Ethics Committee (CONEP123/98). Kaingang and Guarani, the two major Amerindian tribes of southern Brazil, are culturally quite distinct from each other, the Guarani belonging to the Tupi linguistic group, while Kaingang are Gê-speaking. The KRC and GRC live in different villages within the Rio das Cobras reservation (25°18′S, 52°32′W), whereas GKW are from the Amambai and Limão Verde reservations (23°06′S, 55°12′W and 23°12′S, 55°06′W, respectively). The HLA-A haplotypes of the Kaingang and Guarani enrolled in the present study have been previously characterized (15 -17; Petzl-Erler and Tsuneto, personal communication).

### Genotype Data From 1 KG_AMR and HGDP American Samples

Aligned exome sequencing from the 1 KG Consortium was retrieved at http://ftp.1000genomes.ebi.ac.uk/vol1/ftp/technical/working/20140725_hla_genotypes/, filtered for 1 KG_AMR superpopulation. Similarly, aligned whole genome sequence of individuals from the Americas included in the HGDP were retrieved at https://www.internationalgenome.org/data-portal/data-collection/hgdp. Reads aligned to chromosome 6, aligned to any of the HLA-I contigs of the GRCh38 reference genome, or classified as unmapped were filtered to ensure a maximum coverage for the HLA-I *loci*. Read filtering, cram to bam conversion and merging were handled by using Samtools 1.13. Class I HLA alleles were identified running Optitype version 1.3.3 (Szolek et al., 2014) with default parameters for DNA input. Individual rs17179220 genotypes were extracted from the HGDP and 1000 Genomes Project databases.

### rs17179220 Genotyping

The TaqMan assay (C_33415939_10) was used in a 7500 Real-Time System for allele discrimination at rs17179220 in the Kaingang and Guarani samples.

### Linkage Disequilibrium and Tag Predictive Parameters

The performance of rs17179220 and rs1061235 as HLA-A*31:01 tags was assessed by the sensitivity, specificity, positive predictive value (PPV) and negative predictive value (NPV) of carriage of the variant SNP alleles (in hetero or homozygosis) to identify carriers of HLA-A*31:01 (in hetero or homozygosis). The software C.I. Calculator: Diagnostic Statistics available online at https://www2.ccrb.cuhk.edu.hk/stat/confidence%20interval/Diagnostic%20Statistic.htm was used for estimating the predictive metrics and respective 95% confidence intervals (CI95).

## Results

### 1 KG_AMR and 1 KG_NAT

HLA-A*31:01 frequency and MAF of rs1061235 were higher in the overall 1 KG_AMR superpopulation than in the 1 KG_NAT subcohort, while the opposite was observed for rs17179220 (Table 1). Both rs1061235 and rs17179220 showed perfect sensitivity and NPV for tagging HLA-A*31:01 carriage in the 1 KG_AMR superpopulation, and, consequently in the 1 KG_NAT cohort.

**TABLE 1:** Predictive performance of rs1061235 and rs17179220 for tagging HLA-A*31:01 in 1KGP samples.

Parameter	1 KG_AMR		1 KG_NAT	
Number of samples	347		80	
Frequency HLA-A*31:01	0.05 (0.03–0.07)		0.02 (0–0.05)	
MAF rs1061235	0.13 (0.11–0.17)		0.03 (0.01–0.07)	
MAF rs17179220	0.12 (0.10–0.15)		0.20 (0.11–0.27)	
	rs1061235	rs17179220	rs1061235	rs17179220
Sensitivity	1.00	1.00	1.00	1.00
Specificity	0.96 (0.93–0.98)	0.86 (0.83–0.90)	0.99 (0.96–1.0)	0.66 (0.56–0.77)
PPV	0.70 (0.56–0.83)	0.42 (0.31–0.53)	0.75 (0.32–1.0)	0.10 (0–0.21)
NPV	1.00	1.00	1.00	1.00

1KGP, 1000 genomes project; MAF, minor allele frequency; NPV, negative predictive value; PPV, positive predictive value. 1 KG_AMR, Admixed American superpopulation. 1 KG_NAT, subcohort of 1 KG_AMR comprising the 80 samples with the highest proportion of Native American ancestry. Values in brackets correspond to 95% confidence interval.

In 1 KG_AMR, specificity was nearly complete (0.96) for rs1061235, and somewhat lower for rs17179220 (0.86), whereas PPV ranged between 0.42 (rs17179220) and 0.70 (rs1061235). All false positives for rs1061235 were due to the presence of this SNV in the HLA-A*33:01 haplotype, as previously reported for other populations (Amstutz et al., 2013; He et al., 2015; Buchner et al., 2021). False positives for rs17179220, however, were not linked to HLA-A*33:01.

In 1 KG_NAT, specificity of the tagging SNVs ranged between 0.66 and 0.95, and PPV varied from 0.10 to 0.75, with both lower values for rs17179220. As a subcohort of 1 KG_AMR, all false positives for rs1061235 in 1 KG_NAT resulted from LD with HLA-A*33:01 haplotype.

### HGDP American Cohort

HLA-A*31:01 frequency and rs17179220 MAF were similar (0.20), whereas rs1061235 MAF was lower (0.12). HLA-A*33 haplotypes were not detected in the HGDP samples, and no false positives affected the predictive performance of rs1061235 as tag for HLA-A*31:01 (Table 2). Accordingly, specificity and PPV of rs1061235 were perfect, whereas NPV and sensitivity were incomplete due to false negatives. By contrast, NPV and sensitivity were perfect for rs17179220, with high specificity (0.98) and PPV (0.94).

**TABLE 2:** Predictive performance of rs1061235 and rs17179220 for tagging HLA-A*31:01 in Native American samples.

Parameter	HGDP		KRC	GRC	GKW
Number of samples	61		57	28	18
Frequency HLA-A*31:01	0.20 (0.13–0.28)		0.65 (0.55–0.74)	0.04 (0–0.12)	0.44 (0.28–0.62)
MAF rs1061235	0.12 (0.07–0.19)				
MAF rs17179220	0.20 (0.13–0.28)		0.66 (0.56–0.74)	0.20 (0.10–0.32)	0.58 (0.41–0.74)
	rs1061235	rs17179220	rs17179220	rs17179220	rs17179220
Sensitivity	0.75 (0.53–0.96)	1.00	0.98 (0.94–1.00)	0.00	1.00
Specificity	1.00	0.98 (0.93–1.00)	1.00	0.58 (0.39–0.77)	0.40 (0–0.83)
PPV	1.00	0.94 (0.84–1.00)	1.00	0.00	0.81 (0.62–1.0)
NPV	0.91 (0.83–0.99)	1.00	0.80 (0.45–1.00)	0.88 (0.73–1.00)	1.00

HGDP, human genome diversity project; KRC, Kaingang; GRC and GKW, Guarani groups; MAF, minor allele frequency; NPV, negative predictive value; PPV, positive predictive value. Values in brackets correspond to 95% confidence interval.

### Kaingang and Guarani

Limited amounts of DNA combined with availability of a predesigned TaqMan assay for rs17179220, but not rs1061235, led us to investigate only rs17179220 as tag for HLA-A*31:01 in Kaingang and Guarani samples. The HLA-A*31:01 frequency and rs17179220 MAF varied over wide ranges, respectively, 0.04–0.65 and 0.20–0.66 across the indigenous groups, while the HLA-A*33 haplotype was not detected (Table 2). Of notice, all KRC (*n* = 20) and GKW (*n* = 3) homozygotes for HLA-A*31:01 carried the rs17179220 variant allele in homozygosis, whereas no GKW sample had both rs17179220*A and HLA-A*31:01. These striking differences in linkage disequilibrium were reflected in the predictive performance of rs17179220 as HLA-A*31:01 tag*,* especially sensitivity and PPV, which ranged from zero to 1.0 across the three groups. Specificity ranged from 0.40 to 1.0, while NPV varied between 0.80 and 1.0.

## Discussion

This is the first study of the predictive performance of previously identified SNP tags for HLA-A*31:01 in indigenous populations of the Americas, represented in the HGDP project and recruited from three reservations areas in Brazil. We extended our analysis to the 1 KG_AMR superpopulation—comprised of individuals with Native American, European and African admixed ancestry—and to a subcohort of the 1 KG_AMR, denoted 1 KG_NAT, of individuals with predominant (> 70%) Native ancestry.

The diversity of our study cohorts is reflected in the frequency of the HLA-A*31:01 haplotype, which ranged from 0.02 in 1 KG_NAT to 0.65 in the KRC (Tables 1, 2). Particularly impressive is the extent to which the indigenous groups from Brazil differ in HLA-A*31:01 frequency, first reported by Petzl-Erler and colleagues (Petzl-Erler et al., 1993; Parham et al., 1997). Of notice, the GRC (Guarani) and KRC (Kaingang), in the extreme of the HLA-A*31:01 frequency range (0.04 and 0.65, respectively) live in the same indigenous reservation area in southern Brazil (Rio das Cobras) and despite their proximity, remain linguistically and culturally distinct and are predominantly endogamous (Petzl-Erler et al., 1993; Parham et al., 1997). Large differences in HLA-A*31:01 frequency were also observed between Karitiana (0.54) and Suruí (HLA-A*31:01 absent), two tribes from the Brazilian Amazon represented in the HGDP cohort. However, the small number of individuals of the distinct HGDP American groups, especially Suruí (*n* = 8), are an obvious caveat to intergroup comparisons.

The diversity of the study cohorts is also evident in the MAF of rs1061235 and rs17179220, which varied 3-5 fold across cohorts, and in the predictive performance of these SNVs as tags for HLA-A*31:01. We highlight especially NPV, the predictive metric of primary interest for pharmacogenetic-informed carbamazepine prescription. Both SNVs showed perfect NPV (NPV = 1.0) in 1 KG_AMR and 1 KG_NAT, while in HGDP NPV was perfect for rs17179220, but not for rs1061235 (NPV = 0.91). In the Native populations from Brazil, genotyped only for rs17179220, perfect NPV was observed in GWK, but not GRC (NPV = 0.88) or KRC (NPV = 0.80). Of notice, rs17179220 was not present in the HLA-A*31:01 haplotype in GRC, such that the PPV and specificity were null. Data from previous genotyping of the Kaingang and Guarani samples for HLA-A, B and C haplotypes (Petzl-Erler et al., 1993; Parham et al., 1997) reveal that in KRC, all the eight different HLA-A*31-Bx-Cx haplotypes included the rs17179220 A allele, while the A*24 and the A*02-B*35 and A*02-B*39 haplotypes as well as a few non-HLA-A*31 haplotypes introduced by gene flow from European or African were rs17179220*G. However, of 10 A*02:12-B*51:01:01-C*01:02 KRC haplotypes, eight included the G and two the A allele. In GKW, all the *HLA-*A*31 haplotypes but also all the eight HLA A*02:01-B*40:04-C*03:04 haplotypes carried the rs17179220 A allele.

Prompted by a Reviewer’s suggestion, we extended our analysis of the predictive performance of rs1061235 and rs17179220 as HLA-A*31:01 tags to other admixed populations of the Americas, with predominant European or SubSaharan African, rather than Native ancestry (Table 3). This exercise was applied to a previously defined subcohort [denoted 1 KG_AMR (European)] comprising the 80 1 KG_AMR samples with the largest proportion of European ancestry (Suarez-Kurtz et al., 2020) and to the 80 1 KG_ACB (African Caribbean) samples with the largest proportion of African ancestry (Debortoli et al., 2021). HLA-A*31:01 frequency ranged from 0.01 in the 1 KG_ACB subcohort to 0.06 in 1 KG_AMR (European). Sensitivity and NPV were perfect for both rs1061235 and rs17179220 in 1 KG_AMR (European) and for rs1061235, but not rs17179220, in the 1 KG_ACB subcohort. We emphasize that the small number of individuals carrying the HLA-A*31:01 haplotype in both these subcohorts, especially the 1 KG_ACB (*n* = 2) is a major caveat for quantification of the predictive metrics. Of notice, the rs1061235 SNV was in complete linkage disequilibrium with the HLA-A*33 in the 1 KG_ACB subcohort, and in strong disequilibrium in 1 KG_AMR (European), where four of the five HLA-A*33 carriers had also the rs1061235 variant allele.

**TABLE 3:** Predictive performance of rs1061235 and rs17179220 for tagging HLA-A*31:01 in 1KGP samples.

Parameter	1 KG_AMR (European ancestry)^a^		1 KG_ACB^b^	
Number of samples	80		80	
Frequency HLA-A*31:01	0.06 (0.03–0.10)		0.01 (0.0–0.04)	
MAF rs1061235	0.05 (0.02–0.10)		0.12 (0.07–0.18)	
MAF rs17179220	0.03 (0.01–0.07)		0.01 (0.0–0.03)	
	rs1061235	rs17179220	rs1061235	rs17179220
Sensitivity	1.00	1.00	1.00	0.50 (0.0–1.0)
Specificity	0.95 (0.89–0.99)	0.93 (0.87–0.99)	0.78 (0.69–0.87)	1.00
PPV	0.50 (0.15–0.85)	0.44 (0.12–0.77)	0.11 (0.0–0.24)	1.00
NPV	1.00	1.00	1.00	0.99 (0.96–1.0)

1KGP, 1000 genomes project.

a Subcohort of the 1 KG_AMR superpopulation, comprising the 80 samples with the largest proportion of European ancestry.

b Subcohort of the 1 KG_ACB population, comprising the 80 samples with the largest proportion of African ancestry.

Values in brackets correspond to 95% confidence interval. MAF, minor allele frequency; NPV, negative predictive value; PPV, positive predictive value.

We acknowledge that the low frequency of HLA-A*31:01 in the GRC sample and the low number of individuals of the distinct groups in the HGDP American cohort pose limitations to our study. Practical and ethical difficulties are commonly encountered in recruiting participants from Native American populations, and the pivotal study linking HLA-A*31:01 to carbamazepine-induced cutaneous reaction in Native Americans included only 5 Aboriginal Canadian children (Amstutz et al., 2013). Another potential limitation relates to the use of different methodologies for characterization of HLA haplotypes in the indigenous populations from Brazil compared to the 1 KG and HGDP samples, which may have affected the results.

In conclusion, we present the first assessment of the predictive performance of rs1061235 and rs17179220 for tagging HLA-A*31:01 in cohorts of Native American ancestry. NPV, the metric of primary interest for pharmacogenetic-informed carbamazepine prescription, ranged from 0.80 to 1.0, which supports the notion that these SNPs are not optimal proxies for HLA-A*31:01 across populations with Native American ancestry.

## Data Availability

The datasets presented in this study can be found in online repositories. The names of the repository/repositories and accession number(s) can be found in the article/supplementary material.
